# Clinical Application of Trans-Arterial Radioembolization in Hepatic Malignancies in Europe: First Results from the Prospective Multicentre Observational Study CIRSE Registry for SIR-Spheres Therapy (CIRT)

**DOI:** 10.1007/s00270-020-02642-y

**Published:** 2020-09-21

**Authors:** Thomas Helmberger, Rita Golfieri, Maciej Pech, Thomas Pfammatter, Dirk Arnold, Roberto Cianni, Geert Maleux, Graham Munneke, Olivier Pellerin, Bora Peynircioglu, Bruno Sangro, Niklaus Schaefer, Niels de Jong, José Ignacio Bilbao, On behalf of the CIRT Steering Committee, Jean-Pierre Pelage, Derek M. Manas, Frank T. Kolligs, Samer Ezziddin, Ralph Peters, On behalf of the CIRT Principal Investigators, Thomas Albrecht, Olivier D’Archambeau, Tugsan Balli, Sadik Bilgic, Alan Bloom, Roberto Cioni, Roman Fischbach, Patrick Flamen, Laurent Gerard, Gerd Grözinger, Marcus Katoh, Michael Koehler, Jan Robert Kröger, Christiane Kuhl, Franco Orsi, Murat Ozgun, Peter Reimer, Maxime Ronot, Axel Schmid, Alessandro Vit

**Affiliations:** 1 Department of Radiology, Neuroradiology and Minimal-Invasive Therapy, Klinikum Bogenhausen, Englschalkinger Str. 77, 81925 Munich, Germany; 2 Radiology Unit, Department of Experimental, Diagnostic and Speciality Medicine, Sant’Orsola Hospital, University of Bologna, Via Bagni di Mario 15, 40136 Bologna, Italy; 3 Department of Radiology and Nuclear Medicine, University of Magdeburg, Leipziger Strasse 44, 39120 Magdeburg, Germany; 4 Institut für Diagnostische und Interventionelle Radiologie, Universitätsspital Zürich, Rämistrasse 100, 8091 Zürich, Switzerland; 5 Oncology and Hematology, Asklepios Tumorzentrum Hamburg, AK Altona, Paul-Ehrlich-Str. 1, 22763 Hamburg, Germany; 6 Department of Interventional Radiology, S. Camillo Hospital, Circonvallazione Gianicolense, 85, 00149 Rome, Italy; 7 Radiology, Universitair Ziekenhuis Leuven, Herestraat 49, 3000 Leuven, Belgium; 8 Interventional Oncology, University College London Hospitals NHS Foundation Trust, 250 Euston Road, NW1 2PG London, UK; 9 Interventional Radiology Department, Hôpital Européen Georges Pompidou, 20 rue Leblanc, 75015 Paris, France; 10 Department of Radiology, School of Medicine, Hacettepe University, Sihhiye Campus, 06100 Ankara, Turkey; 11 Liver Unit, Clínica Universidad de Navarra, IDISNA and CIBEREHD, Avda. Pio XII 36, 31008 Pamplona, Spain; 12 Service de médecine nucléaire et imagerie moléculaire, Inselspital Hospital Lausanne, Rue du Bugnon 46, 1011 Lausanne, Switzerland; 13 Clinical Research Department, Cardiovascular and Interventional Radiological Society of Europe, Neutorgasse 9, 1010 Vienna, Austria; 14 Interventional Radiology, Clínica Universidad de Navarra, Avenida Pio XII, No 36, 31008 Pamplona, Spain

**Keywords:** Hepatocellular carcinoma, Metastasis, Observational study, Registries, Therapeutic embolization, Liver, Yttrium-90, Radioisotope brachytherapy, Trans-arterial radioembolization

## Abstract

**Purpose:**

To address the lack of prospective data on the real-life clinical application of trans-arterial radioembolization (TARE) in Europe, the Cardiovascular and Interventional Radiological Society of Europe (CIRSE) initiated the prospective observational study *CIRSE Registry for SIR-Spheres® Therapy (CIRT)*.

**Materials and Methods:**

Patients were enrolled from 1 January 2015 till 31 December 2017. Eligible patients were adult patients treated with TARE with Y90 resin microspheres for primary or metastatic liver tumours. Patients were followed up for 24 months after treatment, whereas data on the clinical context of TARE, overall survival (OS) and safety were collected.

**Results:**

Totally, 1027 patients were analysed. 68.2% of the intention of treatment was palliative. Up to half of the patients received systemic therapy and/or locoregional treatments prior to TARE (53.1%; 38.3%). Median overall survival (OS) was reported per cohort and was 16.5 months (95% confidence interval (CI) 14.2–19.3) for hepatocellular carcinoma, 14.6 months (95% CI 10.9–17.9) for intrahepatic cholangiocarcinoma. For liver metastases, median OS for colorectal cancer was 9.8 months (95% CI 8.3–12.9), 5.6 months for pancreatic cancer (95% CI 4.1–6.6), 10.6 months (95% CI 7.3–14.4) for breast cancer, 14.6 months (95% CI 7.3–21.4) for melanoma and 33.1 months (95% CI 22.1–nr) for neuroendocrine tumours. Statistically significant prognostic factors in terms of OS include the presence of ascites, cirrhosis, extra-hepatic disease, patient performance status (Eastern Cooperative Oncology Group), number of chemotherapy lines prior to TARE and tumour burden. Thirty-day mortality rate was 1.0%. 2.5% experienced adverse events grade 3 or 4 within 30 days after TARE.

**Conclusion:**

In the real-life clinical setting, TARE is largely considered to be a part of a palliative treatment strategy across indications and provides an excellent safety profile.

**Level of evidence:**

Level 3.

**Trial registration:**

ClinicalTrials.gov NCT02305459.

**Electronic supplementary material:**

The online version of this article (10.1007/s00270-020-02642-y) contains supplementary material, which is available to authorized users.

## Introduction

Current guidelines for the treatment of primary liver malignancies (e.g. hepatocellular carcinoma (HCC), intra-hepatic cholangiocarcinoma (ICC)) and hepatic metastases, e.g. from colorectal cancer (mCRC), propose trans-arterial radioembolization (TARE, also known as selective internal radiation therapy (SIRT)) as an optional treatment modality for patients with liver dominant disease not suitable for surgical or ablative therapies, or who experienced no response, significant side effects or intolerance when treated with systemic therapies. [1–7].

At the time of the study’s conception in 2014, available studies on TARE consisted of large cohort series and smaller experimental trials [8–17]. In the meantime, several large-scale randomized controlled trials on TARE in mCRC and HCC have been completed and published [18–22], as well as large prospective and retrospective studies on HCC, ICC and mCRC [23–30]. As more centres in Europe included TARE in their armamentarium of treatments for liver malignancies, there was a need for a multicentre, prospective data collection on the use of TARE in clinical practice beyond high-expertise centres, where countries with different health-care systems were able to contribute to evaluate how TARE is used in standard clinical practice in Europe [31]. A recent multicentre prospective observational study in the UK describes the outcome of TARE in clinical practice for mCRC and ICC [32, 33], and a large-scale prospective observational study on TARE is currently being conducted in the USA (NCT02685631). Physicians and patients will benefit from the insights provided by real-world data from European countries and from patients with other liver malignancies beside HCC, mCRC and ICC. Data on less established uses of TARE such as metastatic liver disease from tumour entities such as breast cancer, malignant melanoma, or pancreatic cancer would be needed to uncover potential benefits of these specific patient groups [34–36].

To further improve the understanding of the real-life clinical application of TARE in Europe, the Cardiovascular and Interventional Radiological Society of Europe (CIRSE) initiated the prospective *CIRSE Registry for SIR-Spheres® Therapy (CIRT)* for patients treated with TARE with Y90 resin microspheres (SIR-Spheres® Y-90 resin microspheres, Sirtex Medical Pty Limited; St. Leonards, NSW, Australia). Besides data on how TARE is embedded in the real-life clinical practice (primary objective), CIRT collected data on safety, effectiveness (overall survival (OS), progression-free survival (PFS), liver-specific PFS and imaging response), quality of life (QOL) and details concerning the treatment application.

This manuscript specifically discusses data concerning real-life application of TARE, therapeutic outcome (in terms of overall survival) and safety (in terms of 30-day mortality and morbidity) for all indications. Future manuscripts will include further analysis of the CIRT data considering, e.g. dosimetry data, PFS, hepatic-PFS, imaging response and QOL, as well as subgroup analyses per indication, including less evaluated indications like liver metastases from NET, breast cancer, pancreatic cancer and melanoma.

## Materials and Methods

### Study Design

CIRT is a prospective, multicentre, single-device, observational study of patients with hepatic malignancies treated with TARE with Y90 resin microspheres as standard of care. As observational study, CIRT did not prescribe or encourage the use of TARE in a particular patient group, but observed its use in the real-life clinical setting. Sites were invited to participate if TARE was in their armamentarium of treatment options to treat hepatic malignancies, and if they met the minimum selection criteria of at least 40 treatments in total, with a minimum of ten procedures within 12 months prior to invitation. From August 2014 to April 2017, 68 sites from 12 countries were invited to participate, of whom 27 included patients, representing five countries in the European Union, Switzerland, Turkey and Israel (see Supplement 1 and Supplement 2).

A detailed manuscript on the methodology of CIRT has been previously reported [37].

Patient inclusion criteria were: the patient was 18 years or older, diagnosed with primary or metastatic liver malignancies, scheduled to be treated with TARE with Y90 resin microspheres. There were no specific exclusion criteria. All included patients signed the informed consent form. Patient recruitment occurred between 1 January 2015 and 31 December 2017. Follow-up data was collected until 31 December 2019; patients were followed up for 24 months or until study exit. Specific follow-up intervals were left to the discretion of the medical teams. It was recommended that patient follow-up data would be collected every 3 months. In case follow-up evaluations were not performed at the site of the TARE treatment, sites were encouraged to obtain follow-up information from referring physicians.

### Assessments

The real-life usage of TARE is determined by evaluating the intention of the treatment per indication, and how the TARE treatment was embedded in between prior and post-interventional hepatic and systemic therapies. Overall survival was measured from day of TARE treatment until date of death. Safety outcomes are described as 30-day morbidity and mortality rates according to the Common Terminology Criteria for Adverse Events, version 5.0. Monitored serious adverse events (SAEs, grade 3 and 4) were abdominal pain, fatigue, fever, nausea, vomiting, gastrointestinal ulceration, gastritis, radiation cholecystitis and radioembolization-induced liver disease (REILD). Patient characteristics, prior treatments and volumetric data were collected around time of treatment. Post-TARE treatments and safety data were collected at every follow-up. Survival status was collected as information became available.

### Bias

As observational study, CIRT is sensitive to selection bias. This was addressed by contractually agreeing with study sites that all consecutive cases would be included. Regular remote monitoring by the CIRSE Clinical Research Department was done to verify if sites included all of their cases and to address missing data and data queries. However, it was not possible to perform source document verification.

### Statistical Analysis

Data regarding the primary endpoint, safety and overall survival (OS) data are presented by summaries and descriptive statistics. Overall survival is presented graphically as Kaplan–Meyer curves and median time-to-event per indication with 95% confidence intervals (CI) being provided. Cox multiple regression is used to assess the impact of the covariates for OS and hazard ratios (HR), 95% CI and *p*-values are provided for all covariates. Covariates were chosen prior to data analysis and were published in a methodology manuscript [37]. *P*-values of < 0.05 are considered statistically significant. Patients who had withdrawn consent or are lost to follow up are censored at the last time they were documented as being alive (OS). All available data are used, and no imputations of missing data are made. Where missing data were observed, it was explained in the summary tables.

All procedures performed were in accordance with the ethical standards of the institutional and/or national research committee and with the 1964 Helsinki Declaration and its later amendments or comparable ethical standards.

## Results

Data from 1050 patients were included in the study. Twenty-three patients were excluded (see Supplement 3). The treated cohort (1027) consisted of 542 (52.8%) patients with primary liver tumours (HCC 422 (41.2%), ICC 120 (11.6%)) and 485 (47.2%) with metastatic liver disease (mCRC 237 (23.1%), neuroendocrine (NET) 58 (5.6%), breast 47 (4.6%) and pancreatic cancer and melanoma 32 each (3.1%), and other metastases 79 (7.7%)). 64.9% of the patients were male, and the median age was 65 years (interquartile range (IQR) 56–72).

### Patient Characteristics and Real-Life Application

#### Primary Liver Tumours

For patients with primary liver tumours, the Eastern Cooperative Oncology Group (ECOG) status was 0 in 58.5% of the patients, with 8.3% having ECOG 2 or higher (Table 1). The presence of ascites was observed in 14.3% of the patients, while cirrhosis was more frequently observed in patients with HCC, 71.1% versus 12.5% in ICC. On the other hand, in patients with ICC, extra-hepatic disease was observed in 30% of the cases versus 9.5% of HCC patients. In patients with HCC, unilateral (right-sided) liver tumours were found in 50.2% of the cases, compared to 27.5% in the ICC cohort, which saw more bilobar liver tumours (59.2%). In total, portal vein thrombosis was found in 30.5% of the cases.

**Table 1:** Patient characteristics–primary tumours

Category	Subcategory	HCC (*n* = 422)	ICC (*n* = 120)	All (*n* = 542)
ECOG status	0–fully active	252 (59.7%)	65 (54.2%)	317 (58.5%)
	1–restricted	136 (32.2%)	41 (34.2%)	177 (32.7%)
	2 or higher	34 (8.1%)	11 (9.2%)	45 (8.3%)
	Missing^b^	–	3 (2.5%)	3 (0.6%)
Extra-hepatic disease	No	382 (90.5%)	84 (70.0%)	466 (86.0%)
	Yes	40 (9.5%)	36 (30.0%)	76 (14.0%)
Ascites	No	357 (84.6%)	107 (89.2%)	464 (85.7%)
	Yes	65 (15.4%)	13 (10.8%)	78 (14.3%)
Cirrhosis	No	122 (28.9%)	105 (87.5%)	227 (41.9%)
	Yes	300 (71.1%)	15 (12.5%)	315 (58.1%)
Location of liver tumours	Bilobar	159 (37.7%)	71 (59.2%)	230 (42.4%)
	Left only	51 (12.1%)	16 (13.3%)	67 (12.4%)
	Right only	212 (50.2%)	33 (27.5%)	245 (45.2%)
Number of liver tumours	1	110 (26.1%)	32 (26.7%)	142 (26.2%)
	2–5	154 (36.5%)	35 (29.2%)	189 (34.9%)
	6–9	23 (5.5%)	10 (8.3%)	33 (6.1%)
	10 or more	55 (13.0%)	10 (8.3%)	65 (12.0%)
	Uncountable	80 (19.0%)	33 (27.5%)	113 (20.8%)
Portal vein	Patent	282 (66.8%)	95 (79.2%)	377 (69.6%)
	Segmental thrombosis	82 (19.4%)	14 (11.7%)	96 (17.8%)
	Lobar thrombosis	38 (9.0%)	7 (5.8%)	45 (8.3%)
	Main thrombosis	20 (4.7%)	4 (3.3%)	24 (4.4%)
Total tumour to liver percentage	Median	9.2%	12.8%	10.0%
	Q1, Q3	3.4%, 20.2%	7.9%, 21.5%	4.4%, 20.4%
	Missing	67 (15.9%)	23 (19.2%)	90 (16.7%)

In the primary liver cancer cohorts, median time from diagnosis to TARE was 188 days (IQR 71–590) for HCC and 201 (IQR 65–468) for ICC (Table 2). 60.0% received TARE with palliative intentions (non-curative, e.g. to prolong freedom from or relief of cancer-related symptoms); tumour downsizing was intended in 29.9% of the cases. In the HCC cohort, prior systemic treatments were provided in 10.7% of the patients, while 44.8% receive some form of prior locoregional treatments such as trans-arterial chemoembolization (TACE) (23.0%) or surgery (17.1%). In contrast, ICC patients received prior systemic treatment in 60.8% of the cases [39.2% received combined regimens based on gemcitabine (see Supplement 4)] and locoregional treatments in 34.2%, primarily in the form of surgical procedures (26.7%). Less than 10% of the primary cancer patients received systemic therapies in a concomitant setting. Following TARE, 31.4% received further systemic treatment: in patients with HCC, 18.9% and 4.5% underwent treatment with a tyrosine kinase inhibitor (TKI) and/or other treatments, respectively. Locoregional treatments were applied in 18.4% of the primary liver cases.

**Table 2:** Patient characteristics—metastatic tumours

Category	Subcategory	mCRC (*n* = 237)	NET (*n* = 58)	Breast (*n* = 47)	Pancreatic (*n* = 32)	Melanoma (*n* = 32)	Other liver metastases (*n* = 79)^a^	All (*n* = 485)
ECOG status	0–fully active	140 (59.1%)	38 (65.5%)	29 (61.7%)	20 (62.5%)	15 (46.9%)	41 (51.9%)	283 (58.4%)
	1–restricted	75 (31.6%)	14 (24.1%)	16 (34.0%)	8 (25.0%)	14 (43.8%)	32 (40.5%)	159 (32.8%)
	2 or higher	18 (7.6%)	5 (8.6%)	2 (4.3%)	3 (9.4%)	1 (3.1%)	6 (7.6%)	35 (7.2%)
	Missing^b^	4 (1.7%)	1 (1.7%)	–	1 (3.1%)	2 (6.3%)	–	8 (1.6%)
Extra-hepatic disease	No	139 (58.6%)	25 (43.1%)	17 (36.2%)	17 (53.1%)	25 (78.1%)	33 (41.8%)	256 (52.8%)
	Yes	98 (41.4%)	33 (56.9%)	30 (63.8%)	15 (46.9%)	7 (21.9%)	46 (58.2%)	229 (47.2%)
Ascites	No	228 (96.2%)	54 (93.1%)	44 (93.6%)	29 (90.6%)	31 (96.9%)	75 (94.9%)	461 (95.1%)
	Yes	9 (3.8%)	4 (6.9%)	3 (6.4%)	3 (9.4%)	1 (3.1%)	4 (5.1%)	24 (4.9%)
Cirrhosis	No	235 (99.2%)	58 (100%)	45 (95.7%)	32 (100%)	32 (100%)	77 (97.5%)	479 (98.8%)
	Yes	2 (0.8%)	–	2 (4.3%)	–	–	2 (2.5%)	6 (1.2%)
Location of liver tumours	Bilobar	158 (66.7%)	51 (87.9%)	37 (78.7%)	23 (71.9%)	32 (100%)	56 (70.9%)	357 (73.6%)
	Left only	23 (9.7%)	–	4 (8.5%)	1 (3.1%)	–	5 (6.3%)	33 (6.8%)
	Right only	55 (23.2%)	7 (12.1%)	6 (12.8%)	8 (25.0%)	–	18 (22.8%)	94 (19.4%)
	Missing	1 (0.4%)	–	–	–	–	–	1 (0.2%)
Number of liver tumours	1	21 (8.9%)	2 (3.4%)	7 (14.9%)	1 (3.1%)	3 (9.4%)	8 (10.1%)	42 (8.7%)
	2–5	52 (21.9%)	9 (15.5%)	4 (8.5%)	6 (18.8%)	5 (15.6%)	12 (15.2%)	88 (18.1%)
	6–9	25 (10.5%)	–	3 (6.4%)	1 (3.1%)	2 (6.3%)	9 (11.4%)	40 (8.2%)
	10 or more	58 (24.5%)	14 (24.1%)	7 (14.9%)	10 (31.3%)	15 (46.9%)	18 (22.8%)	122 (25.2%)
	Uncountable	81 (34.2%)	33 (56.9%)	26 (55.3%)	14 (43.8%)	7 (21.9%)	32 (40.5%)	193 (39.7%)
Portal vein	Patent	234 (98.7%)	58 (100%)	43 (91.5%)	32 (100%)	30 (93.8%)	75 (94.9%)	472 (97.3%)
	Segmental thrombosis	3 (1.3%)	–	3 (6.4%)	–	1 (3.1%)	2 (2.5%)	9 (1.9%)
	Lobar thrombosis	–	–	–	–	1 (3.1%)	1 (1.3%)	2 (0.4%)
	Main thrombosis	–	–	1 (2.1%)	–	–	1 (1.3%)	2 (0.4%)
Total tumour to liver percentage	Median	8.9%	20.8%	7.8%	6.6%	10.7%	10.9%	10.5%
	Q1, Q3	3.8%, 18.3%	8.5%, 40.0%	4.0%, 18.6%	3.8%, 14.0%	5.0%, 18.8%	4.0%, 25.6%	4.5%, 21.8%
	Missing	40 (18.9%)	17 (29.3%)	18 (38.3%)	6 (24.0%)	12 (37.5%)	12 (15.2%)	105 (21.6%)

#### Metastatic Liver Tumours

In patients with metastatic liver tumours, ECOG 0 was observed in 58.4% of the patients (Table 3). Extra-hepatic disease was present in 52.8% of the patients, but ascites and cirrhosis were observed in 4.9% and 1.2% of the patients, respectively. Most of the patients (73.6%) had bilobar tumour burden with a liver to tumour percentage of 10.5%. Portal vein thrombosis was observed in 2.6% of the cases.

**Table 3:** Real-life application—primary tumours

Category	Subcategory	HCC (*n* = 422)	ICC (*n* = 120)	All (*n* = 542)
Time since primary diagnosis (days)	Median	188	201	191
	Q1, Q3	71, 590	65, 468	70, 652
	Missing	4 (0.9%)	2 (1.7%)	6 (0.1%)
Intention of treatment^c^	Ablation	17 (4.0%)	7 (5.8%)	24 (4.4%)
	Bridge to liver surgery	3 (0.7%)	3 (2.5%)	6 (1.1%)
	Bridge to liver transplant	23 (5.5%)	2 (1.7%)	25 (4.6%)
	Downsizing / down-staging	137 (32.5%)	25 (20.8%)	162 (29.9%)
	Palliative	242 (57.3%)	83 (69.2%)	325 (60.0%)
Prior TARE hepatic procedures	Yes	189 (44.8%)	41 (34.2%)	230 (42.4%)
	No	233 (55.2%)	79 (65.8%)	312 (57.6%)
	Surgical (any)^a^	72 (17.1%)	32 (26.7%)	104 (19.2%)
	Ablation (any)	62 (14.7%)	7 (5.8%)	69 (12.7%)
	TACE (any)	97 (23.0%)	2 (1.7%)	99 (18.3%)
	Vascular (any)	15 (3.6%)	1 (0.8%)	16 (3.0%)
	Abdominal radiotherapy (any)	7 (1.7%)	5 (4.2%)	12 (2.2%)
Prior systemic therapy	Yes	45 (10.7%)	73 (60.8%)	118 (21.8%)
	No	377 (89.3%)	47 (39.2%)	424 (78.2%)
Concomitant chemotherapy^b^	Yes	32 (7.6%)	11 (9.2%)	43 (7.9%)
	No	390 (92.4%)	109 (90.8%)	499 (92.1%)
Post-TARE systemic therapy	Yes	125 (29.6%)	45 (37.5%)	170 (31.4%)
	No	262 (62.1%)	63 (52.5%)	325 (60.0%)
	Missing^d^	35 (8.3%)	12 (10.0%)	47 (8.7%)
Post-TARE hepatic procedures	Yes	80 (19.0%)	20 (16.7%)	100 (18.4%)
	No	307 (72.7%)	88 (73.3%)	395 (72.9%)
	Missing^d^	35 (8.3%)	12 (10.0%)	47 (8.7%)
	Surgical (any)^a^	3 (0.7%)	4 (3.3%)	7 (1.3%)
	Ablation (any)	11 (2.6%)	4 (3.3%)	5 (2.8%)
	TACE (any)	34 (8.1%)	1 (0.8%)	35 (6.5%)
	Vascular (any)	7 (1.7%)	2 (1.7%)	9 (1.7%)
	Abdominal radiotherapy (any)	13 (3.1%)	6 (5.0%)	19 (3.5%)

^a^Patients can have multiple prior and post-TARE hepatic procedures

^b^Concomitant if systemic therapy start date is within 4 weeks of first TARE treatment start date and up to 8 weeks after first TARE end date (where end date is within 42 days of first TARE in case of two sessions)

^c^Intention of TARE is for first treatment

^d^Missing data include data from patients that were lost to follow up or deceased before the first follow-up could be included (*n* = 47)

Median time from diagnosis of the liver metastases to TARE was 579 days (IQR 253–1089) for the complete metastatic cohort, ranging between 84 days (IQR 56–315) for melanoma metastases to 1242 days (IQR 441–2196) in NET (Table 4). Similar to the primary liver tumour cohort, the intention of TARE was palliative in 77.3% of the patients and downsizing of the tumour in 15.3%. 88.0% of the patients received systemic treatment, and 33.6% received locoregional treatment prior to TARE. 13.2% of the patients received systemic treatments in a concomitant setting. After TARE, systemic treatment was applied in 35.1% of the patients. 13.8% received locoregional treatments.

**Table 4:** Real-life application—metastatic tumours

Category	Subcategory	mCRC (*n* = 237)	NET (*n* = 58)	Breast (*n* = 47)	Pancreatic (*n* = 32)	Melanoma (*n* = 32)	Other liver metastases (*n* = 79)	All (*n* = 485)
Time since metastatic diagnosis (days)	Median	438	1242	1089	514	84	437	579
	Q1, Q3	230, 785	441, 2196	386, 2297	258, 850	56, 315	281, 877	253, 1089
	Missing	39 (16.5%)	12 (20.7%)	7 (14.9%)	7 (21.9%)	1 (3.1%)	11 (13.9%)	77 (15.9%)
Intention of treatment^c^	Ablation	18 (7.6%)	5 (8.6%)	3 (6.4%)	2 (6.3%)	–	6 (7.6%)	34 (7.0%)
	Bridge to liver surgery	2 (0.8%)	–	–	–	–	–	2 (0.4%)
	Bridge to liver transplant	–	–	–	–	–	–	0 (0.0%)
	Downsizing/down-staging	41 (17.3%)	4 (6.9%)	4 (8.5%)	9 (28.1%)	3 (9.4%)	13 (16.5%)	74 (15.3%)
	Palliative	176 (74.3%)	49 (84.5%)	40 (85.1%)	21 (65.6%)	29 (90.6%)	60 (75.9%)	375 (77.3%)
Prior TARE hepatic procedures	Yes	86 (36.3%)	27 (46.6%)	11 (23.4%)	14 (43.8%)	1 (3.1%)	24 (30.4%)	163 (33.6%)
	No	150 (63.3%)	31 (53.4%)	36 (76.6%)	18 (56.3%)	31 (96.9%)	55 (69.6%)	322 (66.2%)
	Missing	1 (0.4%)	–	–	–	–	–	1 (0.2%)
	Surgical (any)^a^	67 (28.3%)	15 (25.9%)	5 (10.6%)	5 (15.6%)	–	16 (20.3%)	108 (22.2%)
	Ablation (any)	27 (11.4%)	4 (6.9%)	2 (4.3%)	6 (18.8%)	1 (3.1%)	6 (7.6%)	46 (9.5%)
	TACE (any)	3 (1.3%)	2 (3.4%)	2 (4.3%)	1 (3.1%)	–	–	8 (1.6%)
	Vascular (any)	3 (1.3%)	3 (5.2%)	1 (2.1%)	–	–	–	7 (1.4%)
	Abdominal radiotherapy (any)	6 (2.5%)	3 (5.2%)	4 (8.5%)	3 (9.4%)	–	5 (6.3%)	21 (4.3%)
Prior systemic therapy	Yes	226 (95.4%)	47 (81.0%)	47 (100%)	27 (84.4%)	13 (40.6%)	67 (84.8%)	427 (88.0%)
	No	11 (4.6%)	11 (19.0%)	–	5 (15.6%)	19 (59.4%)	12 (15.2%)	64 (12.0%)
Concomitant chemotherapy^b^	Yes	31 (13.1%)	7 (12.1%)	6 (12.8%)	4 (12.5%)	4 (12.5%)	12 (15.2%)	64 (13.2%)
	No	206 (86.9%)	51 (87.9%)	41 (87.2%)	28 (87.5%)	28 (87.5%)	67 (84.8%)	421 (86.8%)
Post-TARE systemic therapy	Yes	87 (36.7%)	16 (27.6%)	20 (42.5%)	7 (21.9%)	12 (37.5%)	28 (35.4%)	170 (35.1%)
	No	106 (44.7%)	34 (58.6%)	21 (44.7%)	19 (59.4%)	17 (53.1%)	42 (53.2%)	239 (49.3%)
	Missing^d^	44 (18.6%)	8 (13.8%)	6 (12.8%)	6 (18.7)	3 (9.4%)	9 (11.4%)	76 (15.7%)
Post-TARE hepatic procedures	Yes	35 (14.8%)	10 (17.2%)	3 (6.4%)	5 (15.6%)	5 (15.6%)	9 (11.4%)	67 (13.8%)
	No	159 (67.1%)	40 (69.0%)	38 (80.9%)	21 (65.6%)	24 (75.0%)	61 (77.2%)	333 (70.5%)
	Missing^d^	43 (18.1%)	8 (13.8%)	6 (12.8%)	6 (18.7)	3 (9.4%)	9 (11.4%)	76 (15.7%)
	Surgical (any)^a^	10 (4.2%)	1 (1.7%)	–	1 (3.1%)	–	–	12 (2.5%)
	Ablation (any)	11 (4.6%)	–	1 (2.1%)	–	1 (3.1%)	1 (1.3%)	14 (2.9%)
	TACE (any)	6 (2.5%)	–	–	–	1 (3.1%)	1 (1.3%)	8 (4.2%)
	Vascular (any)	2 (0.8%)	1 (1.7%)	–	–	3 (9.4%)		15 (1.6%)
	Abdominal radiotherapy (any)	10 (4.2%)	7 (12.1%)	2 (4.3%)	4 (12.5%)	–	4 (5.1%)	27 (5.6%)

^a^Patients can have multiple prior and post-TARE hepatic procedures

^b^Concomitant if systemic therapy start date is within 4 weeks of first TARE treatment start date and up to 8 weeks after first TARE end date (where end date is within 42 days of first TARE in case of two sessions)

^c^Intention of TARE is for first treatment

^d^Missing data include data from patients that were lost to follow up or deceased before the first follow-up could be included (*n* = 76)

### Overall Survival

During the observation period, 495 (48.2%) patients died and 349 (33.9%) were lost to follow up. 26 (2.5%) patients had less than 2 years of follow-up but no recorded reason for non-completion. 157 (15.3%) patients were alive and completed the 2-year follow-up period (see Supplement 5).

**Fig. 1:**
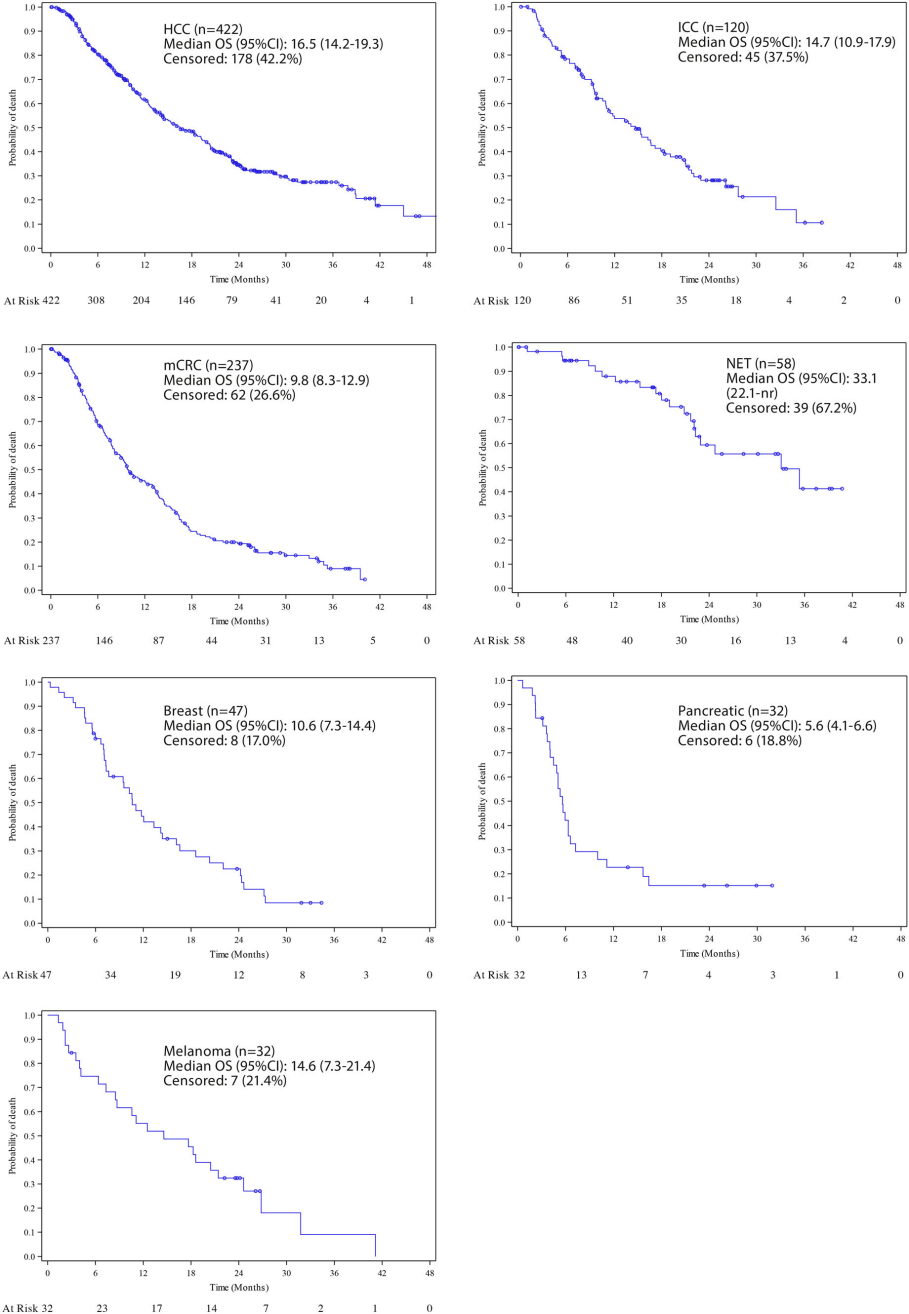
Kaplan–Meier curves per indication of overall survival in months after TARE, including at risk patients per interval

Median overall survival for patients following TARE was 16.5 months (95% CI 14.2–19.3) for HCC and 14.7 months (95% CI 10.9–17.9) for ICC. For liver metastases, median OS for mCRC was 9.8 months (95% CI 8.3–12.9), 5.6 months (95% CI 4.1–6.6) for pancreatic cancer metastases, 10.6 months (95% CI 7.3–14.4) for breast cancer, 14.6 months (95% CI 7.3–21.4) for melanoma and 33.1 months (95% 22.1–nr) for neuroendocrine tumours (see Fig. 1).

For the whole cohort, covariate analyses showed that extra-hepatic disease and ECOG status ≥ 0 were associated with a lower survival rate (HR 1.372, 95% CI 1.149–1.638, *p* < 0.0001; HR 1.513, 95% CI 1.280–1.789, for ECOG 1 and HR 1.624, 95% CI 1.217–2.168 for ECOG 2, *p* < 0.0001, respectively), as well as the presence of cirrhosis (HR 1.304, 95% CI 1.063–1.599, *p* = 0.0128) and ascites (HR 1.344, 95% CI 1.035–1.746, *p* = 0.0039). Unilateral malignancies had a better survival outcome than bilobar malignancies (HR 0.790, 95% CI 0.589–1.059 for left liver lobe tumours and HR 0.694, 95% CI 0.572–0.843 for right liver lobe tumours, *p* = 0.0024), and a higher tumour burden was negatively associated with survival (HR 1.414, 95% CI 1.143–1.750, *p* = 0.0195 was found for a tumour to liver percentage of more than 20%). Prior systemic chemotherapy (yes/no) did not qualify as a significant prognostic factor (*p* = 0.2068); however, the number of chemotherapy lines prior to TARE was found to be statistically significant (increased HR compared to no prior chemotherapy, *p *< 0.0001). Covariates as sex, number of liver tumours and prior hepatic procedures did not produce any significant differences in results (*p*-values > 0.05, see Table 5).

**Table 5:** Covariate analysis

Covariate	Level	Events (%)	HR estimate^a^	95% CI	*p* value^b^
ECOG	0-Fully active	58.7% (352/600)	1.000		
	1-Restricted	73.2% (246/336)	1.513	[1.280, 1.789]	< 0.0001
	2 or higher	70.0% (56/80)	1.624	[1.217, 2.168]	
Extra-hepatic disease prior to treatment	No	59.0% (426/722)	1.000		
	Yes	76.7% (234/305)	1.372	[1.149, 1.638]	< 0.0001
Cirrhosis	No	65.6% (463/706)	1.000		
	Yes	61.4% (197/321)	1.304	[1.063, 1.599]	0.0128
Ascites	No	63.6% (588/925)	1.000		
	Yes	70.6% (72/102)	1.344	[1.035, 1.746]	0.0039
Tumour to liver percentage	Less than 10%	59.0% (242/410)	1.000		
	10%–20%	62.0% (127/205)	1.137	[0.914, 1.413]	0.0195
	Greater than 20%	66.8% (147/220)	1.414	[1.143, 1.750]	
	Unknown	75.0% (144/192)	1.098	[0.879, 1.373]	
Location of liver tumours	Bilobar	71.4% (419/587)	1.000		
	Left only	57.0% (57/100)	0.790	[0.589, 1.059]	0.0024
	Right only	54.3% (184/339)	0.694	[0.572, 0.843]	
Prior chemotherapy: number of lines	0	57.4% (296/516)	1.000		
	1	64.4% (123/191)	1.176	[0.931, 1.485]	< 0.0001
	2–5	76.8% (172/224)	1.855	[1.493, 2.303]	
	6 or more	72.5% (66/91)	1.355	[1.010, 1.818]	

^a^A hazard ratio above 1 implies a higher rate of non-survival for that category compared to the reference category (for which the hazard ratio is 1.000). Selection of covariates based on a stepwise procedure. Variables that did not qualify (*p* > 0.05) were: sex (*p* = 0.2800), prior systemic therapy (*p* = 0.2664), prior hepatic procedures (*p* = 0.0895) and number of liver tumours (*p* = 0.0964)

^b^*P* values are from global Wald test

### Safety

Across the entire cohort, the 30-day mortality rate of patients that received TARE was 1.0% (*n* = 10, (see Supplement 5). Serious adverse events (SAE, grade 3 and 4) within 30 days of treatment were found in less than 2.5% of the patients. SAEs such as gastritis, gastrointestinal ulcerations, radiation cholecystitis and radioembolization-induced liver disease (REILD) occurred in less than 0.3% of the total patient cohort.

## Discussion

The results reported here derive from the largest prospective study on TARE to date and provide a good representation of the European application of TARE in its diverse clinical context. This study provides valuable information on the real-life clinical application and outcomes of TARE in indications for which guidelines are available and used (HCC, ICC, mCRC), as well as insights in the less established use of TARE in liver metastases of NET, breast cancer, pancreatic cancer and melanoma.

The data indicate that in the real-life clinical setting, TARE is largely considered to be a part of a palliative treatment strategy, across indications. That is to say to prolong freedom from or relief of cancer-related symptoms. The relatively low number of patients receiving any systemic therapy (33.1%) or loco-regional treatments (16.3%) after TARE suggests that TARE is used as “last meaningful treatment” rather than being planned as an early consolidation in the scope of various treatment options, suggesting that TARE is used according to most of the current European guidelines [1–5]. Our reported safety data confirming a favourable toxicity profile of TARE may support the consideration of its use earlier in the armamentarium.

Considering the timing of the TARE treatment in relation to prior systemic therapies, our study reported that the majority of the metastatic liver malignancies (mCRC, NET, breast and pancreatic) were treated with TARE after one or more systemic therapy line (Supplement 2). For mCRC, studies have shown that good results can still be achieved in heavily pre-treated patients (see below) [38–40]. In NET, TARE can be considered for patients not responding to systemic therapies or have undergone prior peptide receptor radionuclide therapy (PRRT), TACE or bland embolization, which is reflected in the long median time from metastatic diagnosis to TARE (1242 days, Table 4) [35, 41, 42]. Due to the high OS generally found in NET patients, care should be taken in applying TARE in NET, as treatment-related deaths have been observed in this patient population [43]. For hepatic breast cancer malignancies, all patients in this study were reported to have received prior systemic therapy and most of them received TARE with palliative intent, which have shown to delay progression and decrease tumour size [44–46]. The timing of TARE in pancreatic and melanoma liver metastases is less well understood [47–49]. A Finnish retrospective study on TARE in melanoma patients with hepatic metastases achieved a median OS for TARE of 18.7 months as a first-line treatment compared to chemotherapy (10.5 months), which is reflected in our reported median time from diagnosis to TARE of 84 days [50].

For our primary cohorts, TARE was provided considerably earlier in the treatment pathway (median 188 days (IQR 71–590) for HCC and median 201 days (IQR 65–468) for ICC), suggesting fewer prior hepatic treatments or systemic therapies. Indeed, current guideline recommendations on HCC suggest TARE fairly early in the treatment pathway [4, 7]. For ICC, TARE is recommended after at least 1 line of systemic therapy in locally advanced and metastatic ICC [2]. A recent retrospective study by Bargellini et al. suggests no significant differences between OS between chemotherapy naïve patients and patients who received prior first-line chemotherapy (with and without progression) [30]. A phase 2 trial by Edeline et al. found a median OS of 22 months in chemotherapy naïve patients treated with glass TARE and concomitant chemotherapy, suggesting that administering TARE early in the treatment pathway of unresectable ICC could be beneficial [51]. In our results, prior systemic therapy was provided to 60.8% of the patients, suggesting that sites may have different approaches concerning the place of TARE in the treatment pathway of patients with ICC.

It is encouraging that the median OS for the different cohorts found in our study is consistent with findings of other studies: in mCRC treated with TARE, White et al. reported a pooled weighted OS of 9.6 months (23 studies, *n* = 2517, 95% CI 8.9–10.4) [33], which is consistent with our findings (OS 9.8 months, 95% CI 8.3–12.9). For the smaller cohorts neuroendocrine, breast, pancreatic and melanoma liver metastases, the median OS found in this study were comparable with the median OS found in other studies (breast, a systematic review of 12 studies (*n* = 452) found an OS of 11.3 months [52]; neuroendocrine, a systematic review of 18 studies (*n* = 870) found a median OS of 27.6 months [42]; pancreatic, OS 5.5 months [48]; melanoma, OS 19.9 months [53] and 18.7 months [50]). This supports the fact that the real-life clinical application of TARE in metastatic liver tumours is in accordance with current evidence and strengthens the expectations regarding survival for patients treated with TARE for these indications.

For primary tumours, systematic reviews from Al-Adra et al. and Boehm et al. reported a median OS of 15.5 months (range 7–22.2 months) and 13.9 months (95% CI 9.5–18.3), respectively [54, 55]. Our ICC cohort presented an OS well within the expected range of survival for patients with ICC treated with TARE (14.7, 95% CI 10.9–17.9). For HCC, RCTs such as SARAH, SIRveNIB and SORAMIC found median OS of 8.0, 8.8 and 12.1 months, respectively [18, 22, 56], while retrospective studies found 12.9 and 12.8 months [12, 57]. Our relatively high median OS (16.5 months) can be explained by our high number of Child-Turcotte-Pugh (CTP) A (81.4%) versus CTP B (18.0%) (see supplement 3) paralleling the data presented by Salem et al. [58] and Sangro et al. [29].

Our study confirms previous findings that independent of indication, prognostic factors commonly associated with an increased survival rate are ECOG 0, reduced tumour burden, lack of cirrhosis and ascites, low number of chemotherapy lines prior to TARE and no extra-hepatic disease [16, 57, 59–62]. Kurilova et al. have shown that in mCRC patients in the salvage setting, 1-year OS can range from 10% to 90% based on independent baseline parameters (number of extra-hepatic disease sites, carcinoembryonic antigen, albumin, alanine aminotransferase level, tumour differentiation level and the sum of the two largest tumour diameters) [38]. Damm et al. have developed a scoring system for patients with mCRC consisting of a combination of tumour load, CEA or CA19-9 levels and Karnofsky index to improve patient selection for TARE [39]. In HCC, the presence of portal vein thrombosis has been identified as a negative prognosticator for survival and will be evaluated in a subsequent subgroup analysis [63, 64]. Potential other prognostic factors such as time from (metastatic) diagnosis to treatment and tumour markers were not evaluated at this time.

Limitations of this study are the observational design, whereas potentially important confounding factors could not be controlled. The relatively high number of patients that were lost to follow up can introduce bias regarding the interpretation of OS. A potential explanation might be the fact that TARE requires a comprehensive infrastructure with patients being referred to specialised centres for the treatment while being followed up by their local physician. Follow-up information was in those cases obtained by contacting the referring physician or, if this was not possible, the patient was considered as lost to follow up. While it was outside of the scope of the study to improve the necessary infrastructure for interventional radiology to follow up on their patients, this study provides an opportunity to reflect on the necessity for interventional radiologists to initiate follow-up standards and order relevant imaging after TARE. The CIRSE initiative Standards of Quality Assurance in Interventional Oncology is an initiative to improve quality assurance in interventional oncology, amongst which post-intervention follow-ups and imaging are one of the quality standards [70] Another limitation has been the timing of the study. In the last years, research on TARE has provided insights in the importance of biomarkers, genetic information and tumour absorbed dose on the oncological outcomes [39, 65–69]. As CIRT was designed before these insights were accepted and applied, data on these outcomes have not been included in the objectives of the study. Finally, this analysis did not take into account the potential differences of national guidelines, reimbursement policies and standards of practice.

## Conclusion

This large-scale prospective observational study confirmed that TARE is safe and effective in the real-life clinical setting across various indications. In the real-life clinical setting, TARE is largely considered to be a part of a palliative treatment strategy and less as a component of early consolidation. Real-life OS is comparable to the results from prior clinical trials. Careful patient selection, also in the salvage setting, has been shown to be essential in the treatment liver malignancies with TARE. As new therapies like immune-oncology become available and synergistic treatment concepts get further accepted, TARE will likely become more and more integrated in the standard armamentarium of oncological treatment regimen.

## Electronic supplementary material

Below is the link to the electronic supplementary material.

Supplementary material 1 (PNG 518 kb)

Supplementary material 2 (DOCX 109 kb)

## Abbreviations

CI: Confidence interval
CIRSE: Cardiovascular and Interventional Radiological Society of Europe
CIRT: CIRSE registry for SIR-spheres therapy
ECOG: Eastern cooperative oncology group
HCC: Hepatocellular carcinoma
HR: Hazard ratio
ICC: Intrahepatic cholangiocarcinoma
IQR: Interquartile range
mCRC: Metastatic colorectal cancer
NET: Neuroendocrine tumour
OS: Overall survival
PFS: Progression-free survival
PRRT: Peptide receptor radionuclide therapy
QOL: Quality of life
RCT: Randomized controlled trials
REILD: Radioembolization-induced liver disease
SAE: Serious adverse event
SIRT: Selective internal radiation therapy
TACE: Trans-arterial chemoembolization
TARE: Trans-arterial radioembolization
TKI: Tyrosine kinase inhibitor
