# Supplementary figures and images for: Clinical Application of Trans-Arterial Radioembolization in Hepatic Malignancies in Europe: First Results from the Prospective Multicentre Observational Study CIRSE Registry for SIR-Spheres Therapy (CIRT)
Source: Cardiovasc Intervent Radiol. 2020 Sep 21;44(1):21–35. doi: 10.1007/s00270-020-02642-y (PMC7728645; doi:10.1007/s00270-020-02642-y)

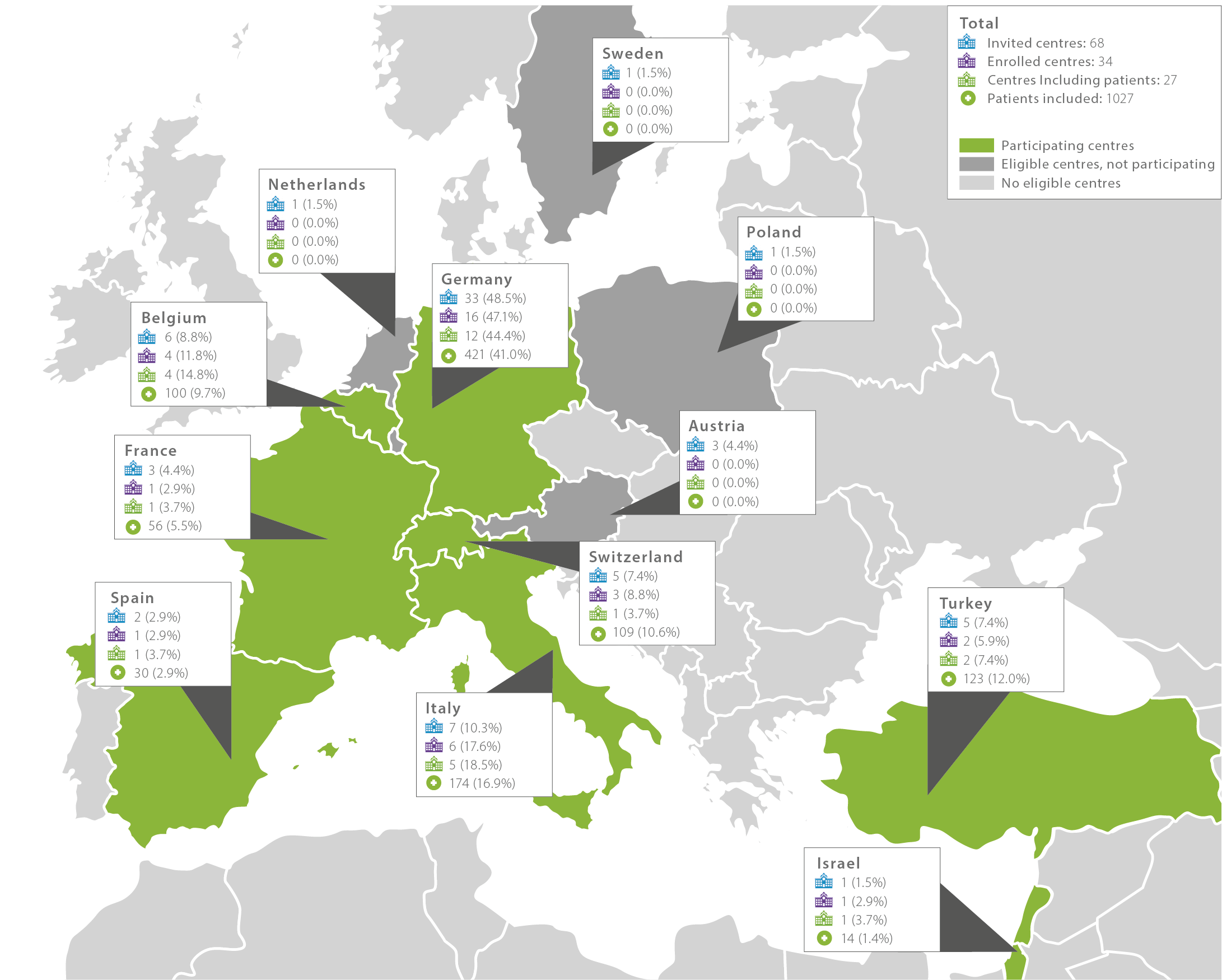

Supplement: Supplementary file 1 — Supplementary material 1 (PNG 518 kb) [file 270_2020_2642_MOESM1_ESM.png]
