# Supplementary material for: Clinical Application of Trans-Arterial Radioembolization in Hepatic Malignancies in Europe: First Results from the Prospective Multicentre Observational Study CIRSE Registry for SIR-Spheres Therapy (CIRT)
Source: Cardiovasc Intervent Radiol. 2020 Sep 21;44(1):21–35. doi: 10.1007/s00270-020-02642-y (PMC7728645; doi:10.1007/s00270-020-02642-y)
Supplement: Supplementary file 2 — Supplementary material 2 (DOCX 109 kb) [file 270_2020_2642_MOESM2_ESM.docx]

Supplementary information

# Supplement 2: distribution of indications per country

| **Category** | **Subcategory** | **HCC (n=422)** | **ICC**  **(n=120)** | **mCRC (n=237)** | **NET (n=58)** | **Breast (n=47)** | **Pancreatic (n=32)** | **Melanoma (n=32)** | **Other liver metastases (n=79)** | **All (n=1027)** |
| --- | --- | --- | --- | --- | --- | --- | --- | --- | --- | --- |
| **Country** | **Germany** | **132 (31.3%)** | **52 (43.3%)** | **111 (46.8%)** | **29 (50.0%)** | **23 (48.9%)** | **19 (59.4%)** | **23 (71.9%)** | **32 (40.5%)** | **421 (41.0%)** |
|  | **Italy** | **113 (26.8%)** | **22 (18.3%)** | **25 (10.5%)** | **-** | **5 (10.6%)** | **2 (6.3%)** | **-** | **7 (8.9%)** | **174 (16.9%)** |
|  | **Turkey** | **56 (13.3%)** | **16 (13.3%)** | **26 (11.0%)** | **5 (8.6%)** | **8 (17.0%)** | **2 (6.3%)** | **-** | **10 (12.7%)** | **123 (12.0%)** |
|  | **Switzerland** | **40 (9.5%)** | **7 (5.8%)** | **22 (9.3%)** | **5 (8.6%)** | **7 (14.9%)** | **2 (6.3%)** | **7 (21.9%)** | **19 (24.1%)** | **109 (10.6%)** |
|  | **Belgium** | **30 (7.1%)** | **4 (3.3%)** | **35 (14.8%)** | **11 (19.0%)** | **3 (6.4%)** | **6 (18.8%)** | **2 (6.3%)** | **9 (11.4%)** | **100 (9.7%)** |
|  | **France** | **40 (9.5%)** | **11 (9.2%)** | **2 (0.8%)** | **3 (5.2%)** | **-** | **-** | **-** |  | **56 (5.5%)** |
|  | **Spain** | **7 (1.7%)** | **6 (5.0%)** | **12 (5.1%)** | **3 (5.2%)** | **-** | **1 (3.1%)** | **-** | **1 (1.3%)** | **30 (2.9%)** |
|  | **Israel** | **4 (0.9%)** | **2 (1.7%)** | **4 (1.7%)** | **2 (3.4%)** | **1 (2.1%)** | **-** | **-** | **1 (1.3%)** | **14 (1.4%)** |

# Supplement 3: CIRT CONSORT Statement


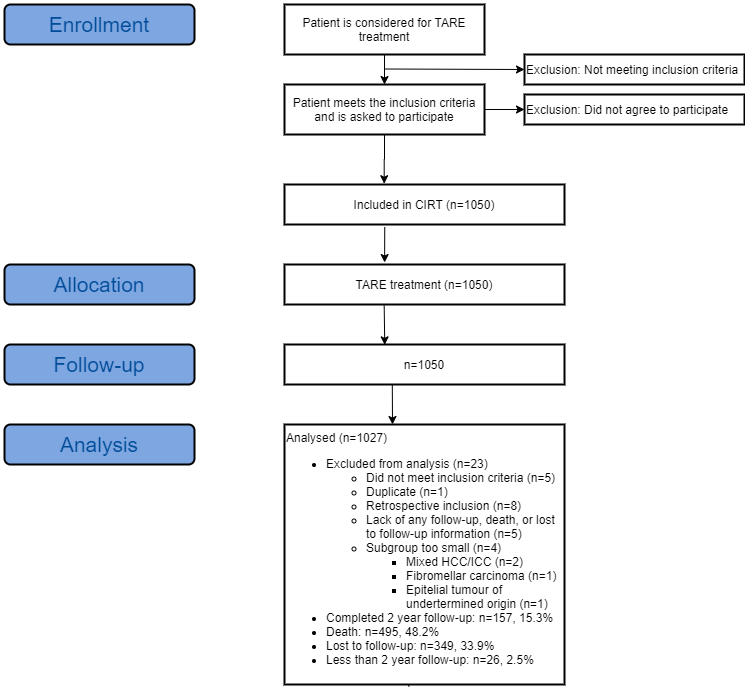


1: CIRT CONSORT Statement

# Supplement 4: Chemotherapies provided prior and post TARE

|  | | | Hepatocellular Carcinoma (n=422) | Cholangio- carcinoma (n=120) | Colorectal Cancer (n=237) | Neuroendocrine Tumor (n=58) | Breast Cancer (n=47) | Pancreatic Cancer (n=32) | Melanoma (n=32) | Secondary/ Metastatic Other  (n=79) | All (n=1027) |
| --- | --- | --- | --- | --- | --- | --- | --- | --- | --- | --- | --- |
| Prior to TARE | Prior systemic therapy | Yes | 45 (10.7%) | 73 (60.8%) | 226 (95.4%) | 47 (81.0%) | 47 (100%) | 27 (84.4%) | 13 (40.6%) | 67 (84.8%) | 545 (53.1%) |
|  |  | No | 377 (89.3%) | 47 (39.2%) | 11 (4.6%) | 11 (19.0%) | - | 5 (15.6%) | 19 (59.4%) | 12 (15.2%) | 482 (46.9%) |
|  | Single or combined (regimens) systemic therapies | n | 45 | 73 | 226 | 47 | 47 | 27 | 13 | 67 | 545 |
|  |  | Combined | 3 (6.7%) | 49 (67.1%) | 172 (76.1%) | 4 (8.5%) | 14 (29.8%) | 14 (51.9%) | - | 24 (35.8%) | 280 (51.4%) |
|  |  | Single | 38 (84.4%) | 13 (17.8%) | 42 (18.6%) | 22 (46.8%) | 25 (53.2%) | 10 (37.0%) | 6 (46.2%) | 30 (44.8%) | 186 (34.1%) |
|  |  | Missing | 4 (8.9) | 11 (15.1%) | 12 (5.3%) | 21 (44.7%) | 8 (17.0%) | 3 (11.1%) | 7 (53.8%) | 13 (19.4%) | 79 (14.5%) |
|  | Number of systemic regimens | n | 3 | 49 | 172 | 4 | 14 | 14 | 0 | 24 | 280 |
|  |  | 1 regimen | 3 (100.0%) | 31 (63.3%) | 71 (41.3%) | 2 (50.0%) | 11 (78.6%) | 8 (57.1%) | - | 13 (54.2%) | 139 (49.6%) |
|  |  | 2 regimens | - | 14 (28.6%) | 51 (29.7%) | 2 (50.0%) | 1 (7.1%) | 4 (28.6%) | - | 5 (20.8%) | 77 (27.5%) |
|  |  | 3-5 regimens | - | 4 (8.2%) | 46 (26.7%) | - | 2 (14.3%) | 2 (14.3%) | - | 5 (20.8%) | 59 (21.1%) |
|  |  | 6-10 regimens | - | - | 4 (2.3%) | - | - | - | - | 1 (4.2%) | 5 (1.8%) |
|  | Combined treatment (multiple treatments per patient possible) | n | 3 | 49 | 172 | 4 | 14 | 14 | - | 24 | 280 |
|  |  | FOLFIRI | - | 9 (18.4%) | 90 (52.3%) | 1 (25.0%) | - | 4 (28.6%) | - | 9 (37.5%) | 113 (40.4%) |
|  |  | FOLFOX | 1 (33.3%) | 4 (8.2%) | 129 (75.0%) | 2 (50.0%) | - | 3 (21.4%) | - | 7 (29.2%) | 146 (52.1%) |
|  |  | FOLFOXIRI | - | - | 12 (7.0%) | - | - | - | - | - | 12 (4.3%) |
|  |  | GEMCIS | - | 37 (75.5%) | - | - | - | 1 (7.1%) | - | 6 (25.0%) | 44 (15.7%) |
|  |  | GEMOX | - | 10 (20.4%) | - | - | - | 1 (7.1%) | - | 2 (8.3%) | 13 (4.6%) |
|  |  | Other | 2 (66.7%) | 1 (2.0%) | 29 (16.9%) | 3 (75.0%) | 14 (100.0%) | 11 (78.6%) | - | 11 (45.8%) | 71 (25.4%) |
|  |  | Not categorised | 1 (33.3%) | 10 (20.4%) | 52 (30.2%) | 3 (75.0%) | 11 (78.6%) | 13 (92.9%) | - | 9 (37.5%) | 99 (35.4%) |
|  | Single systemic therapy | n | 38 (9.0%) | 13 (10.8%) | 42 (17.7%) | 22 (37.9%) | 25 (53.2%) | 10 (31.3%) | 6 (18.8%) | 30 (38.0%) | 186 (18.1%) |
|  |  | Tyrosine kinase inhibitor | 36 (94.8%) | - | - | - | 2 (8.0%) | 1 (10.0%) | - | 4 (13.3%) | 43 (23.1%) |
|  |  | Cytotoxic chemotherapy | 1 (2.6%) | 12 (92.3%) | 42 (100.0%) | 7 (31.8%) | 21 (84.0%) | 9 (90.0%) | 2 (33.3%) | 25 (83.3%) | 119 (63.9%) |
|  |  | Immunotherapy | 1 (2.6%) | - | - | - | - | - | 2 (33.3%) | - | 3 (1.6%) |
|  |  | Octreotide | - | - | - | 10 (45.5%) | - | - | - | - | 10 (5.4%) |
|  |  | Peptide receptor radionuclide therapy | - | - | - | 4 (18.2%) | - | - | - | - | 4 (2.3%) |
|  |  | Antihormonal Therapy | - | - | - | 1 (4.5%) | 1 (4.0%) | - | - | - | 2 (1.1%) |
|  |  | monoclonal antibody (non-immuno-oncology) monotherapy | - | 1 (6.7%) | - | - | 1 (4.0%) | - | 2 (33.3%) | 1 (3.3%) | 5 (2.7%) |
| After TARE | Post-TARE systemic therapy | n ^d^ | 422 | 120 | 237 | 58 | 47 | 32 | 32 | 79 | 1027 |
|  |  | Yes | 125 (29.6%) | 45 (37.5%) | 87 (36.7%) | 16 (27.6%) | 20 (42.5%) | 7 (21.9%) | 12 (37.5%) | 28 (35.4%) | 340 (33.1%) |
|  |  | No | 262 (62.1%) | 63 (52.5%) | 106 (44.7%) | 34 (58.6%) | 21 (44.7%) | 19 (59.4%) | 17 (53.1%) | 42 (53.2%) | 564 (54.9%) |
|  |  | Missing ^d^ | 35 (8.3%) | 12 (10.0%) | 44 (18.6%) | 8 (13.8%) | 6 (12.8%) | 6 (18.7) | 3 (9.4%) | 9 (11.4%) | 123 (12.0%) |
|  | Single or combined (regimens) systemic therapies | n | 125 | 45 | 87 | 16 | 20 | 7 | 12 | 28 | 340 |
|  |  | Combined | 3 (2.4%) | 17 (37.8%) | 24 (27.6%) | 1 (6.3%) | - | 1 (14.3%) | - | 1 (3.6%) | 47 (13.8%) |
|  |  | Single | 99 (79.2%) | 10 (22.2%) | 26 (29.9%) | 6 (37.5%) | 5 (25.0%) | 2 (28.6%) | 7 (58.3%) | 12 (42.9%) | 167 (49.1%) |
|  |  | Missing | 23 (18.4%) | 18 (40.0%) | 37 (42.5%) | 10 (62.5%) | 15 (75.0%) | 4 (57.1%) | 5 (41.7%) | 15 (53.6%) | 126 (37.1%) |
|  | Number of systemic regimens | n | 3 | 17 | 24 | 1 | 0 | 1 | 0 | 1 | 47 |
|  |  | 1 regimen | 2 (66.7%) | 9 (52.9%) | 9 (37.5%) | - | - | - | - | 1 (100.0%) | 21 (44.7%) |
|  |  | 3-5 regimens | - | 5 (29.4%) | 5 (20.8%) | - | - | - | - | - | 10 (21.3%) |
|  |  | 2 regimens | 1 (33.3%) | 2 (11.8%) | 4 (16.7%) | 1 (100.0%) | - | 1 (100.0%) | - | - | 9 (19.1%) |
|  |  | 6-10 regimens | - | 1 (5.9%) | 3 (12.5%) | - | - | - | - | - | 4 (8.5%) |
|  |  | More than 10 regimens | - | - | 3 (12.5%) | - | - | - | - | - | 3 (6.4%) |
|  | Combined treatment (multiple treatments per patient possible) | n | 3 | 17 | 24 | 1 | - | 1 | - | 1 | 47 |
|  |  | FOLFIRI | - | 6 (35.3%) | 18 (75.0%) | 1 (100.0%) | - | - | - | - | 25 (53.2%) |
|  |  | FOLFOX | - | 4 (23.5%) | 7 (29.2%) | - | - | 1 (100.0%) | - | 1 (100.0%) | 13 (27.7%) |
|  |  | FOLFOXIRI | - | - | 1 (4.2%) | - | - | - | - | - | 1 (2.1%) |
|  |  | GEMCIS | - | 4 (23.5%) | - | - | - | - | - | - | 4 (8.5%) |
|  |  | GEMOX | 3 (100.0%) | 6 (35.3%) | - | - | - | - | - | - | 9 19.1% |
|  |  | Other | - | 1 (5.9%) | 3 (12.5%) | - | - | - | - | - | 4 (8.5%) |
|  |  | Not categorised | 3 (100.0%) | 5 (29.4%) | 11 (45.8%) | - | - | 1 (100.0%) | - | 1 (100.0%) | 21 (44.7%) |
|  | Single systemic therapy | n | 99 | 10 | 26 | 6 | 5 | 2 | 7 | 12 | 167 |
|  |  | Tyrosine kinase inhibitor | 80 (80.8%) | 2 (20.0%) | 3 (11.5%) | 1 (16.7%) | - | - | - | 4 (33.3%) | 90 (53.9%) |
|  |  | Cytotoxic hemotherapy | 12 (12.1%) | 8 (80.0%) | 18 (69.2%) | 1 (16.7%) | 4 (80.0%) | 1 (50.0%) | 3 (42.9%) | 7 (58.3%) | 54 (32.2%) |
|  |  | Immunotherapy | 7 (7.1%) | - | - | - | - | - | 3 (42.9%) | 1 (8.3%) | 11 (6.6%) |
|  |  | Octreotide | - | - | - | 4 (66.7%) | 1 (20.0%) | - | - | - | 5 (3.0%) |
|  |  | Peptide receptor radionuclide therapy | - | - | - | - | - | - | - | - | - |
|  |  | Antihormonal Therapy | - | - | - | - | - | - | - | - | - |
|  |  | monoclonal antibody (non-immuno-oncology) monotherapy | - | - | 5 (19.2%) | - | - | 1 (50.0%) | 1 (14.3%) | - | 7 (4.2%) |
| ^d^ Missing data includes data from patients that were lost to follow-up or deceased before the first follow-up could be included (n=122). | | | | | | | | | | | |

# Supplement 5: Safety: 30 days mortality and morbidity rate

|  | | **HCC (n=422)** | **ICC**  **(n=120)** | **mCRC (n=237)** | **NET (n=58)** | **Breast (n=47)** | **Pancreatic (n=32)** | **Melanoma (n=32)** | **Other liver metastases (n=79)** | **All (n=1027)** |
| --- | --- | --- | --- | --- | --- | --- | --- | --- | --- | --- |
| Deceased | Within 30 days* | 3 (0.7%) | 1 (0.8%) | 4 (1.7%) | - | 1 (2.1%) | 1 (3.1%) | - | - | 10 (1.0%) |
| Adverse event Grade 3 or higher | Abdominal Pain | 9 (2.1%) | 4 (3.3%) | 4 (1.7%) | 1 (1.7%) | 3 (6.4%) | 2 (6.3%) | 1 (3.1%) | 1 (1.3%) | 25 (2.4%) |
|  | Fatigue | 6 (1.4%) | 2 (1.7%) | - | 1 (1.7%) | 2 (4.3%) | - | 1 (3.1%) | 2 (2.5%) | 14 (1.4%) |
|  | Fever | 2 (0.5%) | - | - | - | - | - | - | - | 2 (0.2%) |
|  | GI Ulceration | 1 (0.2%) | 1 (0.8%) | 2 (0.8%) | - | - | - | - | - | 4 (0.4%) |
|  | Gastritis | - | 1 (0.8%) | 2 (0.8%) | - | - | - | - | - | 3 (0.3%) |
|  | Nausea | 3 (0.7%) | - | 1 (0.4%) | - | - | - | - | 1 (1.3%) | 5 (0.5%) |
|  | Other | 15 (3.6%) | 8 (6.7%) | 18 (7.6%) | 2 (3.4%) | 2 (4.3%) | 3 (9.4%) | 2 (6.3%) | 1 (1.3%) | 51 (5.0%) |
|  | Radiation Cholecystitis | - | 1 (0.8%) | 1 (0.4%) | - | - | - | - | - | 2 (0.2%) |
|  | Radioembolisation-Induced Liver Disease (REILD) | 3 (0.7%) | 2 (1.7%) | - | - | - | - | - | - | 5 (0.5%) |
|  | Vomiting | 2 (0.5%) | - | - | - | - | - | - | - | 2 (0.2%) |
| *7/10 patients died from intra-hepatic or extra-hepatic disease progression. 1 patient from pleural effusions and ascites. 2 patients exact cause of death unrelated to the treatment or the disease. | | | | | | | | | | |

# Supplement 6 – Child-Turcotte-Pugh (CTP) scores for HCC

|  | | Hepatocellular Carcinoma (n=422) |
| --- | --- | --- |
| Child-Turcotte-Pugh severity (calculated) | n^a^ | 162 |
|  | A | 131 (80.9%) |
|  | B | 30 (18.5%) |
|  | C | 1 (0.6%) |
| Child-Turcotte-Pugh score | n^a^ | 162 |
|  | Mean (SD) | 5.8 (0.9) |
|  | Median | 5.5 |
|  | Q1, Q3 | 5.0, 6.0 |
|  | Min, Max | 5, 10 |
| n^a^: Number of patients available for analyses. | | |
